# Stearoyl-CoA desaturase indexes and n-6/n-3 fatty acids ratio as biomarkers of cardiometabolic risk factors in normal-weight rabbits fed high fat diets

**DOI:** 10.1186/s12929-016-0235-6

**Published:** 2016-01-20

**Authors:** Gabriela Alarcón, Julieta Roco, Analia Medina, Carina Van Nieuwenhove, Mirta Medina, Susana Jerez

**Affiliations:** Instituto Superior de Investigaciones Biológicas (INSIBIO, UNT-CONICET), Av Independencia 1800, San Miguel de Tucumán, Tucumán 4000 Argentina; Facultad de Ciencias Naturales e Instituto Miguel Lillo, Universidad Nacional de Tucumán (UNT), Miguel Lillo 205, San Miguel de Tucumán, Tucumán 4000 Argentina; Centro de Referencias para Lactobacilos (CERELA, CONICET), Chacabuco 145, San Miguel de Tucumán, Tucumán 4000 Argentina

**Keywords:** Metabolic syndrome, High fat diet, Stearoyl-coA desaturase, n-6/n-3 fatty acid ratio, Biomarkers, Visceral abdominal fat

## Abstract

**Background:**

Biomarkers for cardiometabolic risk (CMR) factors would be important tools to maximize the effectiveness of dietary interventions to prevent cardiovascular diseases. Thus, the aim of this work was to analyze stearoyl-CoA desaturase (SCD) indexes and n-6/n-3 fatty acids (FA) ratio as biomarkers of CMR induced by feeding rabbits on high fat diets (HFDs).

**Methods:**

Rabbits were fed either regular diet or 18 % fat in regular diet (HFD) or 1 % cholesterol diet (HD) or diet containing 1 % cholesterol and 18 % fat (HFD-HD) during 6 weeks. Body weights (BW), blood pressure, visceral abdominal fat (VAF) and glucose tolerance test were determined. Total cholesterol (TC), low density lipoprotein-cholesterol (LDL-C), high density lipoprotein-cholesterol (HDL-C), triglycerides (TG), fasting glucose (FG), and FA levels from plasma were measured. SCD indexes were calculated as product/precursor ratios of individual FA.

**Results:**

BW was similar in all diet groups. HD increased TC, LDL-C, HDL-C, and TG. HFD increased TG, VAF and FG, and decreased HDL-C. The addition of HFD to HD joined to dyslipidemia increased VAF and FG. SCD indexes were increased and n-6/n-3 was unchanged in HD. SCD indexes were reduced and n-6/n-3 FA ratio was increased in HFD and HFD-HD. CMR factors were correlated positively with n-6/n-3 FA ratio. Although VAF had a stronger correlation with n-6/n-3 FA ratio than with SCD indexes, VAF was associated independently to both markers.

**Conclusions:**

HFD simulating lipid composition of the average Western-style diet induced experimental rabbit models of normal-weight metabolic syndrome (MS). SCD indexes and n-6/n-3 were modified according to the type of dietary fat. Considering that VAF and CMR factors appear to be stronger associated to n-6/n-3 FA ratio than to SCD indexes, n-6/n-3 FA ratio may be a better biomarker of MS and CMR in normal-weight subjects than SCD indexes.

## Background

It is well recognized that the dietary fat is strongly related with the development of cardiovascular disease. According to the Consensus Conference Report published by the American Diabetes Association and the American College of Cardiology Foundation [1], cardiometabolic risk (CMR) refers to a high lifetime risk for cardiovascular disease. Metabolic syndrome (MS) is a cluster of metabolic alterations characterized by systemic alterations that may involve several organs and tissues: abdominal obesity, glucose intolerance, hyperinsulinemia, hypertriglyceridemia, decreased levels of high density lipoprotein-cholesterol (HDL-C) and hypertension [2]. CMR is similar to MS but is more inclusive, as it also includes other risk factors such as high levels of total cholesterol (TC) and low density lipoprotein-cholesterol (LDL-C). Dietary saturated fat intake has been shown to increase the risk of heart disease and stroke. It is estimated to cause about 31 % of coronary heart disease and 11 % of stroke worldwide.

A biomarker is defined as a measurable variable that may be used as an indicator of a given biological state or condition [3]. Biomarkers have been used for diagnostic purposes or therapeutic interventions, or as risk markers for predicting the development of certain pathologies. Given the close association of MS with fat diets [4], validation of biomarkers is needed to test the efficacy of diet interventions or novel treatments in progressive MS. Despite decades of attempting to identify sensitive and specific biomarkers for diagnosis, prognosis, and treatment efficacy of MS, no generally accepted markers or laboratory tests have emerged as of yet.

The delta-9 desaturase enzymes (also referred to as stearoyl-CoA desaturases, SCD) catalyze the rate limiting step in the conversion of saturated (SFA) to monounsaturated fatty acids (MUFA) (mainly oleic acid [OA, 18:1 n-9] and palmitoleic acid [16:1 n-7] from stearic acid [18:0] and palmitic acid [16:0], respectively). Oleic and palmitoleic acids are the major MUFA in fat depots and membrane phospholipids. The ratio of stearic acid to OA, estimated activity of SCD-18, is one of the factors influencing membrane fluidity and cell–cell interaction [5]. Abnormal alteration of this ratio has been shown to play a role in several pathological states including diabetes, cardiovascular diseases, obesity, hypertension, neurological diseases, immune disorders, cancer, and aging. High activities of estimated SCD-16 (as the ratio of palmitic acid to palmitoleic acid) and SCD-18 have been associated with obesity and dietary fat. Thus, SCD indexes may predict the development of MS [6, 7].

Today’s Western-style diets are characterized by increases in total fat intake, especially in saturated fat and n-6 fatty acids, and decreases in n-3 fatty acids in comparison to the fatty acids (FA) intake during evolution for which our genes were programmed to respond [8]. Population-based intervention studies reveal contradictory results. Some evidence suggests that increases in the n-3/n-6 FA ratio might be effective in reducing insulin resistance and the prevalence of MS [9, 10]. Other studies suggest no link between different dietary intake of n-3 FA and n-6 FA and the prevalence of MS [11]. Guptan et al. [12] have recently demonstrated that serum n-6/n-3 FA ratio may be a risk marker for cardiovascular diseases.

Rabbits are animals suitable as tools for translational research. Various high fat diets (HFD) and intervention periods have been used to induce MS in rabbits or its components like insulin resistance, visceral obesity, hypertension, and dyslipidemia [13]. In a previous work, we have characterized a model of MS by feeding rabbits on a 10 % HFD during 12 weeks [14].

Considering that biomarkers for MS and CMR may be important tools to test the efficacy of dietary interventions in reducing cardiovascular diseases, this article will mainly focus on the analysis of SCD-16 and SCD-18 indexes and the n-6/n-3 FA ratio as biomarkers related to the CMR factors induced by feeding rabbits on HFDs.

## Methods

### Animal handling and diets

Experiments were reviewed and approved by our Institutional Animal Care and Use Committee (Bioethics Committee of the School of Medicine of the National University of Tucuman, Argentina). Thirty two male hybrid rabbits (Cabaña Los Prietos, Las Talitas, Tucumán, Argentina), weighing 800–900 g on arrival, were individually housed in metal cages in a room with controlled temperature, humidity, and a 12-h light cycle. After one week acclimation period, animals were randomly assigned to four groups of eight animals. The four diets included a control diet (CD) of regular rabbit chow (Ganave, Pilar, Buenos Aires, Argentina), a 1 % cholesterol (Sigma Chemical, St Louis, Missouri, USA)-supplemented chow (HD), a 18 % fat-supplemented chow (HFD), and a diet supplemented with 1 % cholesterol and 18 % fat (HFD-HD). The excess fat in the diet consisted of corn oil (10 %) and lard (8 %). The diets were prepared by mixing the regular rabbit chow with the added components. After thorough mixing, the diets were re-pelleted and allowed to dry in a well-ventilated area. Rabbits were fed a restricted amount (100 g/day) of the respective diet for 6 weeks. Dietary composition is detailed in Table 1. Only male rabbits were used to avoid secondary variability related to sex differences in these experimental models.

**Table 1:** Composition of experimental diets (g/100 g diet)

	CD	HD	HFD	HFD-HD
Carbohydrates	34	34	34	34
Protein	15	15	15	15
Fiber	15	15	15	15
Total fat	3	3	18	18
Fatty acids				
14:0 (Miristic acid)	0.27 ± 0.1	0.20 ± 0.01	0.29 ± 0.01	0.26 ± 0.02
C16:0 (Palmitic acid)	18.9 ± 0.1	16.7 ± 0.47	21.25 ± 0.35	20.3 ± 0.28
C18:0 (Stearic acid)	1.62 ± 0.3	2.32 ± 0.13	2.4 ± 0.14	2.02 ± 0.14
Total SFA	21.4 ± 0.1	19.2 ± 0.5	23.9 ± 0.35	22.6 ± 0.28
16:1 n-7 (Palmitoleic acid)	0.13 ± 0.1	0.13 ± 0.01	0.22 ± 0.03	0.28 ± 0.02
18:1 n-9 (Oleic acid)	17.2 ± 0.1	14.3 ± 0.37	22.15 ± 0.35	21.75 ± 0.29
Total MUFA	17.6 ± 0.1	15.6 ± 0.4	22.37 ± 0.3	22.1 ± 0.3
18:2 n-6 (Linoleic acid)	50.0 ± 0.5	60.2 ± 0.42	49.4 ± 0.14	51.3 ± 0.56
18:3 n-3 (Linolenic acid)	6.3 ± 0.04	6.35 ± 0.21	4.51 ± 0.13	4.06 ± 0.35
Total PUFA	56.7 ± 0.5	66.6 ± 0.4	53.9 ± 0.14	55.36 ± 0.56
Total calories (Kcal/100)	250	250	412	412

*SFA* saturated fatty acids, *MUFA* monounsaturated fatty acids, *PUFA* polyunsaturated fatty acids

### Hemodynamics and biochemical assessment

An intraperitoneal glucose tolerance test (GTT) was performed two days before the end of the 6 weeks of feeding as was previously described [14]. The animals were deprived of food, but not of water for 12 h during the night. The GTT was performed on the next morning through intraperitoneal injection of 2 g/kg glucose. Blood samples were obtained from the ear vein before glucose injection (0 min) and at min 60 and min 120 after glucose injection. The blood concentrations of glucose were measured immediately after blood collection with a glucometer (Accu-chek^®^ Active, Mannheim, Baden-Württemberg, Germany) based on the glucose oxidase method using one drop of whole blood.

At the end of the dietary intervention, food was withdrawn for 12 hours, and the rabbits were weighed. Animals were anesthetized with ketamine (20 mg/kg) and diazepam (0.5 mg/kg). Mean blood pressure (MAP) was measured directly in the carotid artery through a catheter connected to a pressure transducer (Gould Instruments, Cleveland, Ohio, USA) and recorded using a data acquisition system (Biopac MP100, Aero Camino Goleta, California, USA). After MAP measurement, blood samples were collected in prechilled glass tubes containing EDTA 10^−7^ M through the catheter inserted in the carotid artery. Using surgical techniques a midline incision was made in the rabbit and adipose tissues from the abdominal areas were collected and weighted. The visceral abdominal fat (VAF) was expressed as a percentage of the total body weight: (fat weight/animal weight) x 100.

Plasma TC, HDL-C, LDL-C, triglycerides (TG), and fasting glucose were measured using colorimetric reactions with commercial kits (Wiener, Rosario, Santa Fe, Argentina).

### Plasma fatty acids analysis

Total lipids were extracted from the plasma using chloroform/methanol solution (2:1, v/v). They were derivated with HCl/methanol solution according to Van Nieuwenhove et al. [15]. One micro liter of fatty acid methyl esters (FAMEs), dissolved in hexane, was injected into a gas chromatograph (GC, Model 6890 N, Agilent Technologies, Wilmington, Delaware, USA) equipped with a flame ionization detector (Agilent Technologies) and an automatic injector (Model 7683, Agilent Technologies, Shanghai, China) into an HP-88 capillary column (100 m × 0.25 mm × 0.20 μm, Agilent Technologies). GC conditions involved an injector with a temperature of 255 °C. The initial oven temperature of 75 °C was increased to 165 °C at 8 °C/min and was held there for 35 min. It was then increased to 210 °C at 5.5 °C/min and maintained for 2 min, and afterwards to 240 °C at 15C/min and held for 3 min. The detector temperature was 280 °C. Nitrogen was used as a carrier gas at a flow rate of 18 mL/min at 38 psi. FAMEs were identified and quantified by comparison of retention times and peak areas with the methylated standards (F.A.M.E. mix, C8-C24; Sigma Chemical), using heptadecanoic acid (C17:0) as internal standard. Results were expressed as g/100 g of FAME.

### Estimation of desaturase activity and n-6/n-3 fatty acids ratio

The desaturase index was calculated as product/precursor ratios of individual FA. In this study, desaturase indexes were estimated as follows: SCD-16 = 16:1 n-7/16:0 and SCD-18 = 18:1 n-9/18:0.

The n-6/n-3 FA ratio was calculated as linoleic acid (LA, 18:2 n-6) plus arachidonic acid (ARA, 20:4 n-6) acids/linolenic acid (ALA, 18:3 n-3) plus docosahexaenoic acid (DHA, 22:6 n-3) plus eicosapentaenoic acid (EPA, 20:5 n-3).

### Statistical analysis

Values are given as mean ± standard error. The differences in the mean values between the four diet groups were tested by two way ANOVA followed by a Duncan’s test. Pearson’s correlation coefficients were carried out to assess relationships between normally distributed variables. The relationships between either SCD indexes or n-6/n-3 FA ratio and CMR factors (TC, HDL-C, LDL-C, TG, fasting glucose, VAF, MAP) were analyzed by multiple linear regression analysis. Forward based stepwise regression procedure was used. A value of *p* < 0.05 was considered statistically significant.

## Results

### Clinical characteristics

HD significantly affected the animal body weight (*p* < 0.01) while HFD did not (*p* = 0.13). In addition no interaction was found (two-way ANOVA and Duncan’s post test). However, HD did not modify VAF with respect to CD while 18 % fat addition to the diet displayed markedly increases of VAF in all diets groups (Table 2).

**Table 2:** Plasma levels of lipids, glucose and body weight values from rabbits fed a control diet (CD), a CD supplemented with 18 % fat (HFD), a high cholesterol diet (HD), a HD supplemented with 18 % fat (HFD-HD)

	CD	HD	HFD	HFD-HD
Body Weight (g)	2138 ± 82	1896 ± 96	2043 ± 46	1845 ± 48
Visceral abdominal fat (g)	0.29 ± 0.05	0.4 ± 0.05	2.07 ± 0.2^a^	1.44 ± 0.1^a^
Fasting Glucose (mg/dl)	98.5 ± 3.2	108.9 ± 4.7	132.8 ± 8.8^a^	148 ± 6^a^
Blood pressure (MAP) (mmHg)	62.7 ± 5.1	75.8 ± 2.3	62 ± 7.1	77.4 ± 9
Total Cholesterol (mg/dl)	59.0 ± 6.0	872.3 ± 114^a^	64.7 ± 4.8	1793 ± 97^a,b^
LDL-cholesterol (mg/dl)	23.8 ± 3.1	666 ± 99^a^	27.1 ± 3.4	1391 ± 108^a,b^
HDL-cholesterol (mg/dl)	58.8 ± 4.1	164 ± 45^a^	29.4 ± 5.7^a^	197 ± 38^a^
Triglycerides (mg/dl)	92 ± 14	222 ± 33^a^	155.6 ± 40^a^	253 ± 27^a^

Data are expressed as mean ± SE of 8 rabbits. ^a^ *P* < 0.05 indicates statistically significant differences between rabbits fed a HFD and HFD-HD with respect to rabbits fed a CD. ^b^ *P* < 0.05 indicates statistically significant differences between rabbits fed a HFD-HD and rabbits fed a HD (two way ANOVA and Duncan’s post test)

**Fig. 1:**
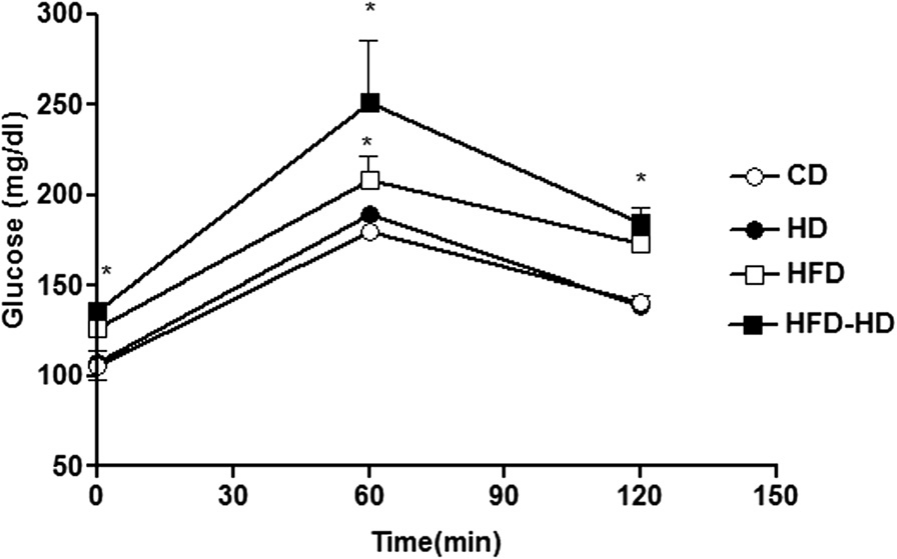
Plasma glucose levels at base line (0 minutes) and at indicated time points after injection of 2 g/kg of glucose from rabbits fed a control diet (CD) and rabbits fed a high cholesterol diet (HD), high fat diet (HFD) or HFD supplemented with 1 % cholesterol (HFD-HD). Data are mean ± SE of 8 animals. *P <0.05 indicates statistically significant differences compared with arteries from rabbits fed a CD

Feeding either the HFD or the HFD-HD significantly increased fasting glucose (Table 2). In addition, glucose tolerance was impaired (Fig. 1). At the end of the experiment, the MAP of the rabbits fed a HD and a HFD-HD was about 20 % higher than that of the other diets groups. Two-way ANOVA analysis showed that HD affect blood pressure (*p* < 0.05) and HFD did not (*p* = 0.3) (Table 2). There was a significantly correlation between fasting glucose levels and VAF (r = 0.68, *n* = 12, *p* <0.05). In addition, glucose levels at 60 minutes after intraperitoneal injection of glucose were positively related to VAF (r = 0.79, *n* = 12, *p* <0.05).

### Effects on lipid concentrations

At the end of the 6 week period of dietary treatment, plasma levels of TC and LDL-C were significantly increased in animals fed high cholesterol diets. Moreover, TC and LDL-C were higher in rabbits fed a HFD- HD than rabbits fed a HD (Table 2). HDL-C was reduced in the HFD group as compared with the other diet groups. The TC/HDL-C ratio, related to metabolic indices predictive of ischemic heart disease risk and insulin resistance syndrome [16], was higher in rabbits fed a HFD-HD than rabbits fed a HD (7.0 ± 0.5 vs 5.3 ± 0.2; *p* < 0.05, unpaired t test). Plasma TG levels were elevated in all fat diets groups (Table 2).

Major FAs present in total FA of the plasma are listed in Table 3. The total levels of SFA were increased and the total levels of MUFA were reduced after the HFD and HFD-HD. Moreover, significant differences in some SFA and MUFA were determined. Relative to the other diets groups, proportion of palmitic acid and palmitoleic acid were higher, while proportion of stearic acid was lower after the HD. Relative to the CD group, proportion of OA was lower after the HD, HFD and HFD-HD. Even more, proportions of OA were reduced after the HFD and the HFD-HD with respect to rabbits fed a HD. Levels of PUFA were increased after the HFD-HD as compared with all diets groups. Proportion of ALA was significantly lower after the HFD. Addition of cholesterol to the HFD potentiated ALA reduction (Table 3). Level of LA and the n-6/n-3 FA ratio was increased in rabbits fed a HFD and HD-HFD as compared with rabbits fed a CD or a HD. Addition of cholesterol to the HFD increased the n-6/n-3 FA ratio with respect to HFD.

**Table 3:** Plasma fatty acids (expressed as % total fatty acids)

	CD	HD	HFD	HFD-HD
Satured fatty acids (SFA)				
16:0 (Palmitic acid)	22.5 ± 0.9	29.8 ± 1.1^a,b^	22.9 ± 0.5	24.1 ± 0.3
18:0 (Stearic acid)	15.1 ± 0.5	8.3 ± 0.4^a,b^	18.7 ± 0.03^a^	19.5 ± 0.08^a^
Total SFA	37.6 ± 0.8	38.1 ± 0.2	41.6 ± 0.5^a^	43.6 ± 0.3^a^
Monounsaturated fatty acids (MUFA)				
16:1 n-7 (Palmitoleic acid)	1.0 ± 0.3	3.0 ± 0.2^a,b^	0.7 ± 0.06^a^	0.34 ± 0.03^a,c^
18:1 n-9 (Oleic acid)	27.7 ± 1.8	21.0 ± 0.8^a^	14.8 ± 0.2^a^	10.6 ± 0.2^a,c^
Total MUFA Polyunsaturated fatty acids (PUFA)	28.7 ± 0.1	24.0 ± 0.5	15.5 ± 0.2^a^	11 ± 0.2^a,c^
18:3 n-3 (Linolenic acid)	1 ± 0.14	1.8 ± 0.3	0.7 ± 0.03^a^	0.3 ± 0.002^a,c^
22:6 n-3 (Docosahexaenoic acid)	1.97 ± 0.07	2.1 ± 0.3	Not-detected	Not-detected
20:5 n-3 (Eicosapentaenoic acid)	0.24 ± 0.02	0.3 ± 0.05	Not-detected	Not-detected
18:2 n-6 (Linoleic acid)	26.4 ± 1.0	25.5 ± 2.0	34 ± 0.8^a^	37.4 ± 0.6^a,c^
20:4 n-6 (Arachidonic acid)	3.0 ± 0.2	6.3 ± 0.3^a,b^	4.8 ± 0.4	4.7 ± 0.4
Total PUFA	32.4 ± 0.5	36.0 ± 0.5	34.8 ± 0.8	42.3 ± 0.6^a,c^
n-6/n-3 ratio	9.2 ± 1.1	7.6 ± 0.6	48.8 ± 0.8^a^	140 ± 0.6^a,c^
SCD-16	0.044 ± 0.01	0.1 ± 0.01^a,b^	0.03 ± 0.005	0.014 ± 0.002^a^
SCD-18	1.83 ± 0.02	2.53 ± 0.02^a,b^	0.79 ± 0.01^a^	0.54 ± 0.03^a,c^

Data are expressed as mean ± SE of 8 rabbits. ^a^ *P* < 0.05 indicates statistically significant differences with respect to rabbits fed a CD. ^b^ *P* < 0.05 indicates statistically significant differences between rabbits fed a HD and the other diet groups. ^c^ *P* < 0.05 indicates statistically significant differences between rabbits fed a HFD-HD and the other diet groups (two-way ANOVA and Duncan’s post test)

SDC-16 and SDC-18 indexes were increased in rabbits fed a HD with respect to the CD group. HFD did not modify SDC-16 but reduced SDC-18 index as compared with the CD. Cholesterol addition to HFD reduced both SDC-16 and SDC-18 index with respect to the other diets groups. A strong negative association was found between LA levels and SCD-16 (*r* = −0.82, *p* < 0.01) and SCD-18 (*r* = −0.92, *p* < 0.001). SCD correlated negatively with VAF and fasting glucose (Table 4). Stepwise multivariate regression analysis showed that SCD was independently and negatively associated to the abdominal fat (R^2^ = 0.52, *p* < 0.01). The association to SCD-18 (β = −0.62, *p* < 0.0001) was stronger than the association to SCD-16 (β = −0.02, *p* < 0.05).

**Table 4:** Pearson correlation coefficients between plasma SCD-16, SCD-18 and n-6/n-3 fatty acids ratio and visceral abdominal fat, fasting glucose, total cholesterol, LDL-cholesterol, HDL-cholesterol, triglycerides and mean arterial blood pressure (MAP)

	SCD-16	SCD-18	n-6/n-3 FA ratio
Visceral abdominal fat (g)	−0.35 *P* < 0.001	−0.46 *P* < 0.001	0.13 *P* = 0.012
Fasting glucose (mg/dl)	−0.14 *P* = 0.015	−0.18 *P* = 0.0013	0.25 *P* < 0.001
Cholesterol (mg/dl)	−0.045 *P* = 0.22	−0.1 *P* = 0.38	0.61 *P* < 0.001
LDL-cholesterol (mg/dl)	−0.08 *P* = 0.12	−0.06 *P* = 0.21	0.59 *P* < 0.001
HDL-cholesterol (mg/dl)	−0.004 *P* = 0.92	−0.004 *P* = 0.90	0.26 *P* < 0.001
Triglycerides (mg/dl)	−0.002 *P* = 0.97	−0.004 *P* = 0.91	0.21 *P* < 0.001
MAP (mmHg)	0.013 *P* = 0.39	0.06 *P* = 0.06	0.009 *P* = 0.51

*P* < 0.05 was statistically significant

Partial correlation coefficients showed negatively associations between n-6/n-3 FA ratio and SCD-16 (*r* = −0.52, *p* < 0.001) or SCD-18 (*r* = −0.69, *p* < 0.001) and positively with several CMR factors (Table 4). Notably, stepwise multivariate linear regression analysis (R^2^ = 0.83, *p* < 0.001) revealed that n-6/n-3 FA ratio was only independently and positively associated with the abdominal fat (β = 25.65, *p* < 0.001) and TC (β = 0.17, *p* < 0.05).

## Discussion

The present study showed that feeding rabbits with a HFD during a short time induced models of dyslipidemia characterized by multiple risk factors compatibles with the definition of MS: excess of fat in the abdominal visceral area, high TG level, low HDL-C level, high fasting glucose level and glucose intolerance. Moreover, the addition of cholesterol to the HFD joined high TC, TC/HDL-C ratio and LDL-C levels to the risk factors increase. Accumulation of VAF but not total weight increase was induced by HFD intake. Therefore, body weight may not be a good marker of adiposity. Considering that in previous work [14] obesity was found in rabbits after 12 weeks of HFD intake, these results would mean that any longer administration of the HFD may be require to induce weight increase. Normal-weight individuals (BMI < 25 kg/m2) who are at increased risk for metabolic disorders like type 2 diabetes can be considered to be “metabolically unhealthy normal-weight” (MUH-NW) or “metabolically obese normal-weight”. This phenotype was first mentioned in the 1980's by Rudermann et al. [17], who also proposed them to be characterized by hyperinsulinemia, insulin resistance, and hypertriglyceridemia, parameters that might also separate metabolically unhealthy from metabolically healthy obesity [18]. Molero-Conejo et al. [19] showed that an inappropriate diet and low physical activity might be responsible for the high insulin levels and dyslipidemias in lean children and adolescents. Regarding to carbohydrates metabolism, HFD and HFD-HD increased fasting glucose and plasma glucose levels after 60 min and 120 min of the glucose injection as compared with a CD. The mechanisms of these abnormalities are not completely known, but previous studies reported that visceral adipose tissue of obese mice and humans may increase free FA influx through the portal vein into the liver, which could induce insulin resistance [20]. According to these authors, a significant correlation between VAF and fasting glucose or glucose levels after 60 min of the glucose injection was found in the present study. Increase of the TG level was found in rabbits fed a HD, HFD and HFD-HD. Furthermore, hypertriglyceridemia was independent of the type of fat added to the diet.

This is the first study to report reduction of SCD indexes in models of CMR induced by a controlled intervention with HFD and HFD-HD. These results do not support earlier data from the bibliography about SCD activity increase in conditions like diabetes, obesity and MS [21, 22]. Chao et al. [23] report that MUFA and SCD activity are associated with alteration of fasting glycemic status and so may be useful markers for assessing type 2 diabetes and CMR. According to that, other authors demonstrated higher SCD activity and plasma palmitoleic acid levels in people with MS compare with those without it [24, 25]. In contrast with these results, the present work showed reduction of total MUFA levels and negative association between SCD index and fasting glucose. Even more, the present work demonstrated that SCD index was negatively and independently associated to VAF. This result was opposed to the Warensjö et al. [6] findings in a human population. Some authors reported that PUFA decrease SCD activity in rats [26] and humans [27], with possible reduction in the body fat. Considering that the present study showed increased LA plasma levels in rabbits feed a HFD and a HFD-HD, and higher total PUFA in the HFD-HD group with respect to all diet groups, a reduction of SCD index induced by high levels of LA may be hypothesized. Previous findings of Jeffcoat and James [28] coupled with the strong and negative correlation found in the present study between LA levels and SCD activity would support this view. Moreover, reduced LA levels together with an increase of SCD activity were found in individuals with characteristics of MS [23, 24]. However, in agreement with other works performed in rats [29, 30], increase of the SCD activities was found in rabbits fed a HD. These results indicate that the regulation of the SCD activity depends of the circulating type of fat and this is associated to the diet. In the present work a controlled dietary intervention was performed while most human studies sampling is based on the clinical characteristics of the subjects. The lack of associations between SCD activities and most of the CMR studied would imply that determinations of SCD activities are not good markers of MS. According to that, recently Matthan et al. [31] demonstrate no correlation between SCD and cardiovascular risk factors.

As was stated in introduction, n-6 and n-3 FA are functionally distinct and their metabolites often have the opposing physiologic functions. While diets rich in n-6 FA are generally pro-inflammatory and promote insulin resistance, the converse is true for n-3 FA enriched diets [32]. Corresponding to Western diets, HFD-induced MS models are enriched for SFA and n-6 PUFA. The present study showed LA (18:2 n-6) increased both in HFD and HFD-HD while ALA (18:3 n-3) plasma levels were reduced by about 30 % in HFD and 70 % in HFD-HD. These results imply that n-6/n-3 FA ratio was increased in rabbits fed a HFD and even more in rabbits fed a HFD-HD. Nigam et al. [33] report a significant relationship between n-6 plasma FA levels and insulin resistance in coronary patients with and without MS. Furthermore, the present work showed that n-6/n-3 FA ratio correlated positively with most of the CMR studied: VAF, fasting glucose, TC, HDL-C, LDL-C, and TG. These results agree with Gupta et al. [12]. However, the multivariate regression analysis showed that only VAF was positively and independently associated to n-6/n-3 FA ratio. Considering that MS is a complex systemic disease in which a key and potentially unifying pathogenetic role is proposed for abdominal obesity [34], n-6/n-3 FA ratio would be a potentially useful biomarker of MS.

## Conclusions

The present study demonstrated that a HFD rich in SFA, n-6 PUFA and cholesterol simulating a Western-style diet induced rabbit models of MS with normal-weight. In such models n-6/n-3 FA ratio and SCD indexes were modified according to the type of dietary fat. SCD indexes were reduced in rabbits with MS and increased in rabbits fed a HD. N-6/n-3 FA ratio was higher in rabbits fed a HFD and a HFD-HD. In opposite to SCD indexes, n-6/n-3 FA ratio was associated with the most of the CMR factors studied. Although VAF was correlated positively and independently to both markers, the association with the n-6/n-3 FA ratio was stronger than the association with the SCD indexes. Furthermore, present results do not support the inhibition of SCD as therapeutic target for MS and allow inferring that n-6/n-3 FA ratio may be a valuable biomarker of MS and CMR.

## Abbreviations

ALA: Linolenic acid
ARA: Arachidonic acid
BW: Body weight
CD: Control diet
CMR: Cardiometabolic risk
DHA: Docosahexaenoic acid
EPA: Eicosapentaenoic acid
FA: Fatty acid
FAME: Fatty acid methyl ester
FG: Fasting glucose
GTT: Intraperitoneal glucose tolerance test
HDL-C: High-density lipoprotein cholesterol
HD: Hypercholesterolemic diet
HFD: High fat diet
LA: Linoleic acid
LDL-C: Low-density lipoprotein cholesterol
MAP: Mean arterial pressure
MS: Metabolic syndrome
MUFA: Monounsaturated fatty acids
OA: Oleic acid
PUFA: Polyunsaturated fatty acids
SFA: Saturated fatty acid
SCD: Stearoyl CoA desaturase
TC: Total cholesterol
TG: Triglycerides
VAF: Visceral abdominal fat
